# Circulating miRNA-155 as a Potential Biomarker for Coronary Slow Flow

**DOI:** 10.1155/2018/6345284

**Published:** 2018-06-25

**Authors:** Qiang Su, Huafeng Yang, Lang Li

**Affiliations:** Department of Cardiology, The First Affiliated Hospital of Guangxi Medical University, Nanning 530021, China

## Abstract

**Objective:**

Recent studies have demonstrated that miRNA-155 is involved in the occurrence and development of atherosclerosis. Furthermore, miRNA-155 has emerged as a new indirect marker for inflammation associated with adverse outcomes in oncology and cardiovascular diseases. This study investigated the correlation between the levels of miRNA-155 and coronary slow flow (CSF).

**Methods:**

A total of 66 patients with CSF and 66 patients with normal coronary flow were enrolled in this study. Coronary flow velocity was determined using the thrombolysis in myocardial infarction frame count (TFC) method. The plasma levels of miRNA-155 were quantified using real-time quantitative polymerase chain reaction.

**Results:**

The plasma levels of miRNA-155 were significantly higher in the CSF group compared to the control group (*P* < 0.05). In addition, miRNA-155 levels were positively correlated with TFC and high-sensitivity C-reactive protein (hs-CRP) levels (*P* < 0.05 for both parameters). Multivariate linear regression analysis demonstrated that plasma miRNA-155 (OR = 2.384, 95% confidence interval 1.847–3.273, *P* = 0.032) and hs-CRP (OR = 1.273, 95% confidence interval 1.036–2.253, *P* = 0.013) were independent predictors for CSF. Using plasma miRNA-155 levels as the test variable, ROC curve analysis indicated that the area under the curve was 0.782 (*P* < 0.05).

**Conclusion:**

Patients with CSF have higher plasma levels of miRNA-155, and this may play an important role in the pathogenesis of CSF, and an elevated plasma miRNA-155 level may be a predictor for CSF. A large-scale and multicenter study is required to elucidate the role of miRNA-155 as a potential biomarker for patients with CSF.

## 1. Introduction

Coronary slow flow (CSF) refers to the phenomenon that in the absence of significant lesions (stenosis <40%) by coronary angiography, there is a delayed perfusion in distal blood flow [1]. The incidence of CSF is 1%~7% in patients undergoing routine coronary angiography, while the incidence of CSF increases to 25% in patients undergoing coronary angiography due to chest pain [2, 3]. About 80% of patients with CSF have recurrent or long-term symptoms such as precordial discomfort, chest pain, or angina. Although several clinical studies have demonstrated that long-term prognosis of the majority of patients with CSF tends to be benign, a small number of patients can develop acute myocardial infarction and sudden death [4, 5]. At present, the exact mechanism of CSF is unclear. Recent studies have demonstrated that dysfunction of vascular endothelium, early-stage atherosclerosis, inflammatory responses, microangiopathy, and abnormal blood components may be involved in the pathophysiology of CSF [6, 7]. Currently, coronary angiography is still the only effective means of detecting CSF. However, due to the high cost, invasive procedure, and test contradictions like iodine allergy, there is a critical need to investigate an inexpensive, simple, and feasible alternative to diagnose CSF at an early stage.

miRNAs are a group of endogenous noncoding single-stranded RNAs that can regulate gene expression and are found in eukaryotes. miRNAs are about 18~25 nucleotides in length and play important roles in biological processes such as differentiation, proliferation, apoptosis, and inflammation [8, 9]. miRNA-155 is a typical multifunctional miRNA, which has been associated with the occurrence and development of atherosclerosis by regulating the functions of CD4+ T lymphocytes, monocytes/macrophages, endothelial cells, and vascular smooth muscle [10, 11]. Due to the lack of relevant studies on the correlation between miRNA-155 levels and CSF, we investigated whether miRNA-155 could be used as a new clinical diagnostic biomarker for CSF.

## 2. Materials and Methods

### 2.1. Study Subjects

A total of 132 patients who underwent coronary angiography at the First Affiliated Hospital of Guangxi Medical University from January 2012 to January 2017 were enrolled in this study. All these patients had no significant vascular lesions (stenosis of lumen diameter < 40%). Sixty-six patients were selected who had a number of frames wherein the contrast agent passed through at least one coronary artery greater than 27 frames by TIMI frame count method or corrected TIMI frame count method [12] and were enrolled as the CSF group. The control group consisted of sixty-six patients who did not meet the above-mentioned criteria. Patients with the following diseases or symptoms were excluded from the study: myocardial infarction, valvular heart disease, all motion abnormalities with left ventricular ejection fraction below 50%, recent acute coronary syndrome (<3 months), recent myocardial infarction history, positive troponin results, previous revascularization history, uncontrolled hypertension (systolic blood pressure > 160 mmHg or diastolic blood pressure > 105 mmHg), severe liver and kidney insufficiencies, autoimmune disease, and tumor or other severe systemic diseases. This study was conducted according to the principles expressed in the Declaration of Helsinki. This study was approved by the Ethics Committee of the First Affiliated Hospital of Guangxi Medical University.

### 2.2. Specimen Collection

Peripheral venous blood was collected in ethylenediaminetetraacetic acid (EDTA) tubes (BD, Franklin Lakes, NJ, USA). Plasma was isolated within 1 hr using centrifugation at 1900 ×g for 10 min at 4°C to remove blood cells and then at 16,000 ×g for 10 min at 4°C to remove additional cellular nucleic acids attached to cell debris. Plasma samples were transferred to RNase/DNase free tubes and stored at −80°C until analyzed.

### 2.3. miRNA Analysis by Real-Time PCR

Total RNA in plasma was isolated using miRNeasy Plasma Kit (QIAGEN, Germany) following the manufacturer's instructions. First-strand cDNA was synthesized using PrimeScript RT reagent kit (Takara, Japan) from 2 *μ*L of total RNA. Quantitative real-time PCR was performed using the StepOnePlus system (Applied Biosystems, America) with SYBR Premix Ex Taq (Takara, Japan). The stem-loop reverse transcription primers and PCR primers for the miRNA-155 (mature miRNA sequence UUAAUGCUAAUCGUGAUAGGGGUU) of interest were purchased from Ribobio (Guangzhou, China). The miRNA-155 expression values were normalized to snRNA U6 expression and were calculated using the 2^–ΔΔCt^ relative quantification method.

### 2.4. Coronary Angiography and CSF Diagnosis

Coronary angiography was performed on all patients with the standard Judkins technique in multiple projections. At least 4 views of the left coronary artery and 2 views of the right coronary artery (RCA) were performed during coronary angiography. Lohexol was used as a contrast agent in all patients. The thrombolysis in myocardial infarction (TIMI) frame count (TFC) was used to quantitatively measure coronary blood flow using a cineangiography at 30 frames per second. The initial frame count was defined as the frame in which concentrated dye occupies the full width of the proximal coronary artery lumen, touching both borders of the lumen, and forward motion down the artery. The final frame was designated when the leading edge of the contrast column initially reached the distal end. The distal end was defined as the distal bifurcation for the left anterior descending artery (LAD), the distal bifurcation of the segment with the longest total distance for the left circumflex artery (LCX), and the first branch of the posterolateral artery for the RCA. CSF was diagnosed using TFC > 27 in at least 1 coronary artery. The LAD frame counts were corrected by dividing with 1.7 to derive the corrected TFC [13]. Three cardiologists who were blinded to the clinical findings independently assessed the TFC.

### 2.5. Statistical Analysis

Normality of distribution was assessed using the Kolmogorov–Smirnov test. Comparisons between the two groups were performed using Fisher's exact test for categorical variables, and Student's *t*-test or Mann–Whitney *U* test was selected for continuous variables. For comparisons between more than two groups, one-way analysis of variance and Tukey–Kramer honestly significant difference tests were used. Pearson chi-square test and Spearman rho test were used to compare qualitative and quantitative variables when appropriate. The correlation between plasma miRNA-155 levels and other variables was assessed using Pearson's correlation coefficient for symmetrically distributed variables and Spearman's correlation coefficient for skewed variables. Univariate and multivariate linear regression analyses were performed to determine the association between clinical variables and miRNA-155 levels. The receiver operating characteristic curve analysis was performed with miRNA-155 to distinguish patients with or without CSF. All tests were two-sided, and *P* < 0.05 was considered statistically significant. All statistical analyses were performed using JMP 10 software (SAS Institute, Inc., Cary, NC, USA).

## 3. Results

### 3.1. Basic Clinical Characteristics of the Study Participants

The CSF group was not significantly different from the control group in terms of age, gender, smoking status, diabetes mellitus, dyslipidemia, blood pressure, body mass index (BMI), family history of coronary artery disease (CAD), and medication use (all *P* > 0.05; Table 1).

### 3.2. TFC and Distribution of Coronary Arteries Involved in CSF

The corrected TFC for LAD, LCX, and RCA and the mean TFC were significantly higher in patients with CSF compared to the control group (*P* < 0.001; Table 2). 50 (75.8%) of 66 CSF patients had CSF in the LAD, 29 (43.9%) of 66 CSF patients had CSF in the LCX, and 32 (48.5%) of 66 CSF patients had CSF in the RCA in this study. Thirty patients had CSF in all three vessels, 17 in two vessels, and 19 in one vessel (Table 2).

### 3.3. Baseline Laboratory Characteristics of the Study Participants

**Figure 1:**
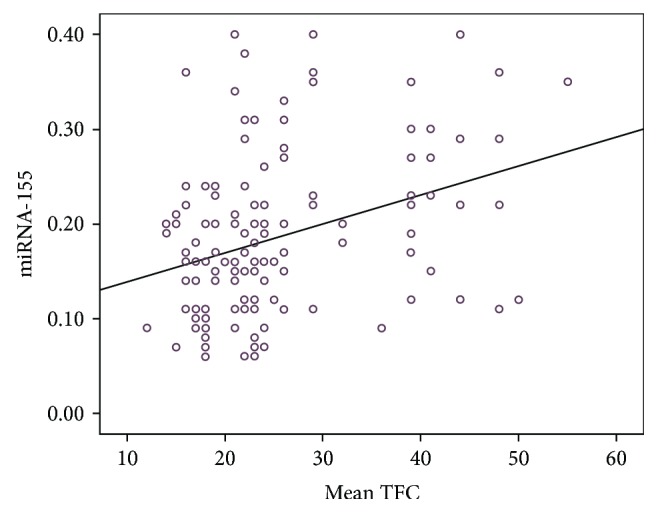
Correlation between mean TIMI frame count (TFC) and miRNA-155 levels.

**Figure 2:**
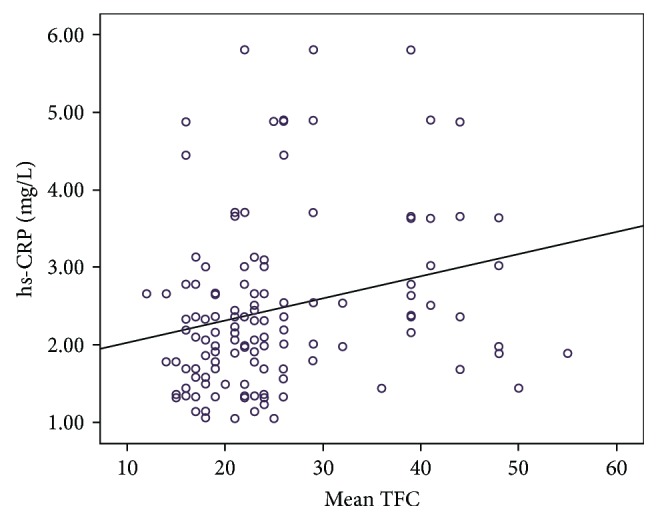
Correlation between mean TIMI frame count (TFC) and hs-CRP levels.

**Figure 3:**
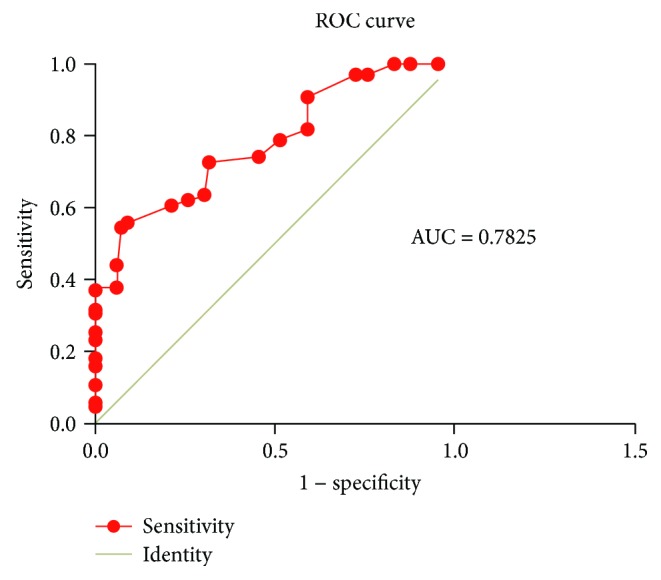
ROC curve of plasma miRNA-155 levels.

Table 3 illustrates the baseline laboratory characteristics of the 2 groups. There were no statistically significant differences between the 2 groups with respect to blood biochemical parameters (Table 3) except for plasma miRNA-155 and high-sensitivity C-reactive protein (hs-CRP) levels. Plasma miRNA-155 and hs-CRP levels were both significantly higher in the CSF group compared to the control group (*P* < 0.001; Table 3). The mean TFC was significantly and positively correlated with plasma miRNA-155 levels (*r* = 0.338, *P* < 0.001; Figure 1) and hs-CRP levels (*r* = 0.247, *P* = 0.004; Figure 2). The ROC curve showed that plasma miRNA-155 levels could be a specific predictor for CSF with an area under the curve of 0.782 (95% confidence interval 0.706–0.859, *P* < 0.001; Figure 3).

### 3.4. Influencing Factors of CSF

**Figure 4:**
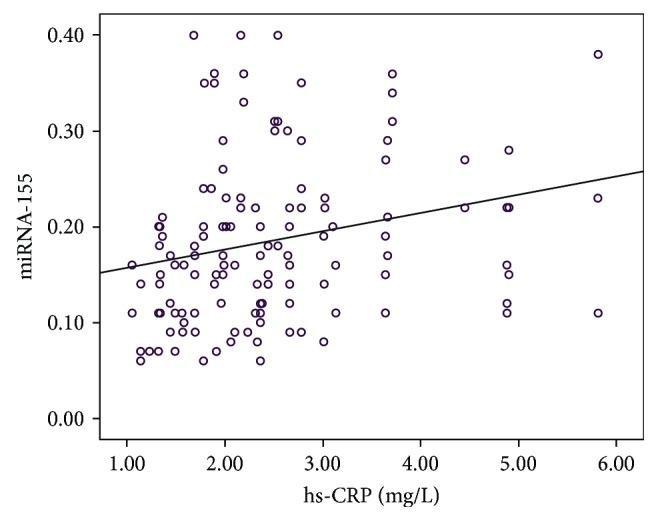
Correlation between miRNA-155 and hs-CRP levels.

Multivariate linear regression analysis showed that plasma miRNA-155 (OR = 2.384, 95% confidence interval 1.847–3.273, *P* = 0.032) and hs-CRP (OR 1.273, 95% confidence interval 1.036–2.253, *P* = 0.013) were independent predictors for CSF (Table 4). Correlation analysis was performed for miRNA-155 and hs-CRP, and the results indicated that there was significant positive correlation between miRNA-155 and hs-CRP (*r* = 0.244, *P* = 0.005; Figure 4).

## 4. Discussion

The major finding of this study was that the plasma miRNA-155 levels were significantly higher in the CSF group compared to the control group and significantly correlated with the mean TFC (an independent predictor of CSF). In addition, miRNA-155 levels were positively correlated with inflammatory markers, hs-CRP. To our knowledge, this is the first study that reports on the relationship between miRNA-155 levels and CSF.

Numerous clinical studies have demonstrated that CSF is the main cause of clinical cardiovascular events such as resting and exertional angina and even myocardial infarction, which severely affects the quality of life in patients [4, 5]. The pathophysiological mechanism of CSF is yet to be deciphered but may be correlated with factors such as microvascular and vascular endothelial dysfunctions, atherosclerosis, inflammatory responses, and abnormal platelet function [6, 7]. Clinicians still lack effective treatment regimens for patients with CSF. Hence, studies on the pathogenesis, pathophysiological process, diagnosis, and treatment of CSF are vital.

miRNAs are a group of small endogenous noncoding RNAs that can degrade mRNA transcripts directly or inhibit protein translation. In addition, they can regulate protein expression at the posttranscriptional level, thereby playing important roles in physiological and pathological processes. A large number of studies have demonstrated that there are numerous miRNAs that are dysregulated in the blood of patients with cancer or cardiovascular diseases compared to healthy controls. These miRNAs regulate the pathophysiological process of organs and tissues [14–16]. Circulating miRNAs are highly stable and will not degrade if stored at room temperature for 24 h or repeatedly freeze-thawed. miRNA expression is tissue-specific and responds rapidly to changes in the body. These characteristics suggest that miRNA can be used as a biomarker for the diagnosis and prognosis of diseases [17]. In recent years, numerous studies have found that miRNAs are closely associated with cardiovascular diseases and play an important role in the diagnosis and prognosis of cardiovascular diseases [18, 19].

miRNA-155 is located in the third exon of the noncoding transcript Bic in human chromosome 21 and was initially named as BIC (B-cell integration cluster). It plays a key role in the immune response and may be involved in the occurrence and development of atherosclerosis by regulating CD4+ T lymphocytes, monocytes/macrophages, endothelial cells, and vascular smooth muscle cells [20]. Zhang et al. [21] demonstrated that miRNA-155 could induce endothelial cell dysfunction by downregulating the expression of endothelial nitric oxide synthase (eNOS), which is involved in the proliferation and migration of vascular smooth muscle cells (VSMCs), thereby promoting atherosclerotic plaque formation. Yang et al. [22] demonstrated that mammalian sterile 20-1ike kinase 2 (MST2) was a target gene of miRNA-155. miRNA-155 can activate the ERK signaling pathway and subsequently aggravated inflammatory responses and oxidative stress response of VSMCs. This promotes the proliferation of VSMCs and vascular reconstruction by inhibiting MST2. In addition, our previous study demonstrated that miRNA-155 was differentially expressed in circulating blood CD4+ T lymphocytes in patients with unstable angina and was involved in the regulation of differentiation and the activation and function of CD4+ T lymphocytes and its subsets [23]. Activated CD4+ T lymphocyte not only secretes IFN-*γ* and activates monocytes but also induces apoptosis of vascular smooth muscle cells and directs the dissolution of endothelial cells by antigen-dependent or antigen-independent pathways, which subsequently leads to the formation of unstable plaques [24]. In this study, we determined that plasma miRNA-155 levels in patients with CSF were significantly higher compared to patients with normal coronary blood flow. The plasma miRNA-155 levels were significantly higher and moderately correlated with the mean TFC. Multivariate logistic regression analysis indicated that plasma miRNA-155 was an independent predictor of CSF. ROC curve analysis demonstrated that the area under the curve was 0.863, suggesting that miRNA-155 was a predictive marker for CSF. These findings suggest that miRNA-155 is an important factor for the manifestation of CSF and may be a good blood biomarker reflecting the state of coronary blood flow.

hs-CRP is a sensitive marker for evaluating low-level inflammation, which is closely related to the occurrence and development of cardiovascular diseases [25]. Wada et al. [26] demonstrated that hs-CRP is an important indicator for risk stratification and risk prediction of acute coronary syndromes and is important for clinical prediction of cardiovascular events. Findings from this study demonstrated that patients with CSF developed hs-CRP symptoms. Stepwise regression analysis was performed for inflammation-associated factors such as age, gender, smoking, alcohol consumption, BMI, and serum lipid levels. We found that hs-CRP was statistically significant in regression analysis, indicating that the mean TFC was positively correlated with plasma hs-CRP. With the increase in hs-CRP levels, blood flow rate slowed, the number of frames increased, and the correlation coefficient was 2.456, suggesting that patients with CSF had hs-CRP symptoms, which promoted inflammatory responses of the coronary artery. Furthermore, we observed dysfunction in the vascular endothelium. In addition, correlation analysis for miRNA-155 and hs-CRP demonstrated significant positive correlation, suggesting that miRNA-155 was closely associated with inflammatory responses in patients with CSF. However, the specific mechanism has yet to be deciphered.

Our study has several limitations. This was a single-center study. We could not demonstrate the mechanisms behind the association between higher miRNA-155 levels in the plasma and CSF. A large-scale and multicenter study is required to elucidate the role of miRNA-155 as a potential biomarker for patients with CSF.

In summary, patients with CSF have higher plasma miRNA-155 levels, and this may play an important role in the pathogenesis of CSF. An elevated plasma miRNA-155 level may indicate the presence of CSF. Further studies are needed to establish the pathophysiological and clinical significance of increased plasma miRNA-155 levels and to investigate the therapeutic efficacy of targeting miRNA-155.

## Figures and Tables

**Table 1:** Basic clinical characteristics of the study participants.

Variable	CSF group (*n* = 66)	Control group (*n* = 66)	*P* value
Male	30 (45.4)	35 (46.7)	0.524
Age (years)	57.18 ± 9.43	55.31 ± 9.82	0.387
BMI (kg/m^2^)	24.2 ± 2.9	23.7 ± 3.3	0.536
Hypertension	22 (33.3)	26 (39.4)	0.216
Diabetes mellitus	12 (18.2)	14 (21.2)	0.182
Dyslipidemia	25 (37.9)	22 (33.3)	0.207
Family history of CAD	13 (19.7)	12 (18.2)	0.341
Current smoker	15 (22.7)	13 (19.7)	0.123
Systolic BP (mmHg)	131.02 ± 15.76	133.24 ± 17.13	0.432
Diastolic BP (mmHg)	77.61 ± 11.23	78.49 ± 12.47	0.533
*Treatment received*			
Aspirin	44 (66.7)	40 (60.6)	0.217
Clopidogrelsulfate bisulfate	22 (33.3)	24 (36.4)	0.312
Statins	36 (54.5)	32 (48.5)	0.094
ACEIs/ARBs	18 (27.3)	16 (24.2)	0.356
Beta blockers	20 (30.3)	23 (34.8)	0.174
Calcium antagonists	14 (21.2)	17 (25.8)	0.105
Nitrates	45 (68.2)	41 (62.1)	0.192

Variables measured with number of patients (percentages in parentheses or mean ± SD). CSF: coronary slow flow; BMI: body mass index; CAD: coronary artery disease; BP: blood pressure; LVEF: left ventricular ejection fraction; ACEIs: angiotensin-converting enzyme inhibitors; ARBs: angiotensin-receptor blockers.

**Table 2:** TFC and distribution of coronary arteries involved in CSF.

Variable	Control group (*n* = 66)	CSF group (*n* = 66)	*P* value
Corrected TFC (LAD)	20.83 ± 3.09	43.00 ± 12.50	<0.001
TFC (LCX)	20.15 ± 3.17	29.14 ± 8.55	<0.001
TFC (RCA)	19.85 ± 3.32	32.08 ± 11.02	<0.001
Mean TFC	19.14 ± 3.23	31.17 ± 10.25	<0.001
One vessel		19 (28.8)	
Two vessels		17 (25.8)	
Three vessels		30 (45.5)	
LAD		50 (75.8)	
LCX		29 (43.9)	
RCA		32 (48.5)	

Variables measured with number of patients (percentages in parentheses or mean ± SD). TFC: TIMI frame count; CSF: coronary slow flow; LAD: left anterior descending artery; LCX: left circumflex artery; RCA: right coronary artery.

**Table 3:** Baseline laboratory characteristics of the study participants.

Variable	Control group (*n* = 66)	CSF group (*n* = 66)	*P* value
WBC (10^9^/L)	5.9 ± 1.2	6.1 ± 1.3	0.3601
Blood glucose	10.42 ± 0.63	10.57 ± 0.58	0.1571
HDL-C (mmol/L)	1.12 ± 0.25	1.18 ± 0.28	0.1964
LDL-C (mmol/L)	2.63 ± 0.73	2.53 ± 0.65	0.4074
TG (mmol/L)	1.48 ± 1.12	1.46 ± 1.16	0.9199
TC (mmol/L)	4.21 ± 0.68	4.24 ± 0.72	0.8060
Serum creatinine (umol/L)	58.62 ± 12.36	58.73 ± 11.43	0.9577
Cystatin C (mg/L)	0.92 ± 0.25	0.87 ± 0.28	0.2812
hs-CRP (mg/L)	1.91 ± 0.61	3.01 ± 1.16	<0.001
miRNA-155	0.09 ± 0.05	0.23 ± 0.09	<0.001

Variables measured with number of patients (percentages in parentheses or mean ± SD). CSF: coronary slow flow; WBC: white blood cells; HDL-C: high-density lipoprotein cholesterol; LDL-C: low-density lipoprotein cholesterol; TG: triglyceride; TC: total cholesterol; hs-CRP: high-sensitivity C-reactive protein.

**Table 4:** Results of logistic regression analysis for factors associated with CSF.

Variable	OR	95% CI	*P* value
Age	0.673	0.564–1.263	0.354
Male	0.454	0.354–1.354	0.437
Hypertension	0.745	0.475–1.236	0.067
Diabetes mellitus	1.236	0.658–2.384	0.352
Current smoker	1.463	0.786–2.374	0.087
Dyslipidemia	0.847	0.674–1.273	0.384
Systolic BP	1.239	0.947–2.384	0.647
Diastolic BP	1.026	0.774–1.748	0.473
HDL-C	0.475	0.384–1.293	0.087
LDL-C	0.748	0.648–1.047	0.064
TG	0.784	0.645–1.293	0.127
TC	0.898	0.746–1.273	0.473
Serum creatinine	0.827	0.792–2.394	0.746
Cystatin C	1.263	0.783–1.348	0.476
BMI	0.764	0.668–2.384	0.087
hs-CRP (mg/L)	1.273	1.036–2.253	0.013
miRNA-155	2.384	1.847–3.273	0.032

CSF: coronary slow flow; OR: odds ratio; CI: confidence interval; BP: blood pressure; HDL-C: high-density lipoprotein cholesterol; LDL-C: low-density lipoprotein cholesterol; TG: triglyceride; TC: total cholesterol; BMI: body mass index; hs-CRP: high-sensitivity C-reactive protein.

## Data Availability

The data used to support the findings of this study are available from the corresponding author upon request.
